# Correction: The violent death toll from the Iraq War: 2003–2023

**DOI:** 10.1371/journal.pone.0306760

**Published:** 2024-07-03

**Authors:** Michael Spagat

In the Discussion section, there is an error in the seventh sentence of the fifth paragraph (last portion of the paragraph). Instead of 200,000 violent deaths, it should be 300,000.

The correct sentence is as follows: These numbers lead to the following tentative conclusions about the human cost of the first 20 years of the war; 1. The IBC-extended figures, roughly 300,000 violent deaths might be 20% higher than the true numbers of violent deaths: 2. The true numbers of violent deaths might, at a stretch, be twice the IBC-extended Fig 3.
