# Supplementary figures and images for: The violent death toll from the Iraq War: 2003–2023
Source: PLoS One. 2024 Feb 27;19(2):e0297895. doi: 10.1371/journal.pone.0297895 (PMC10898744; doi:10.1371/journal.pone.0297895)

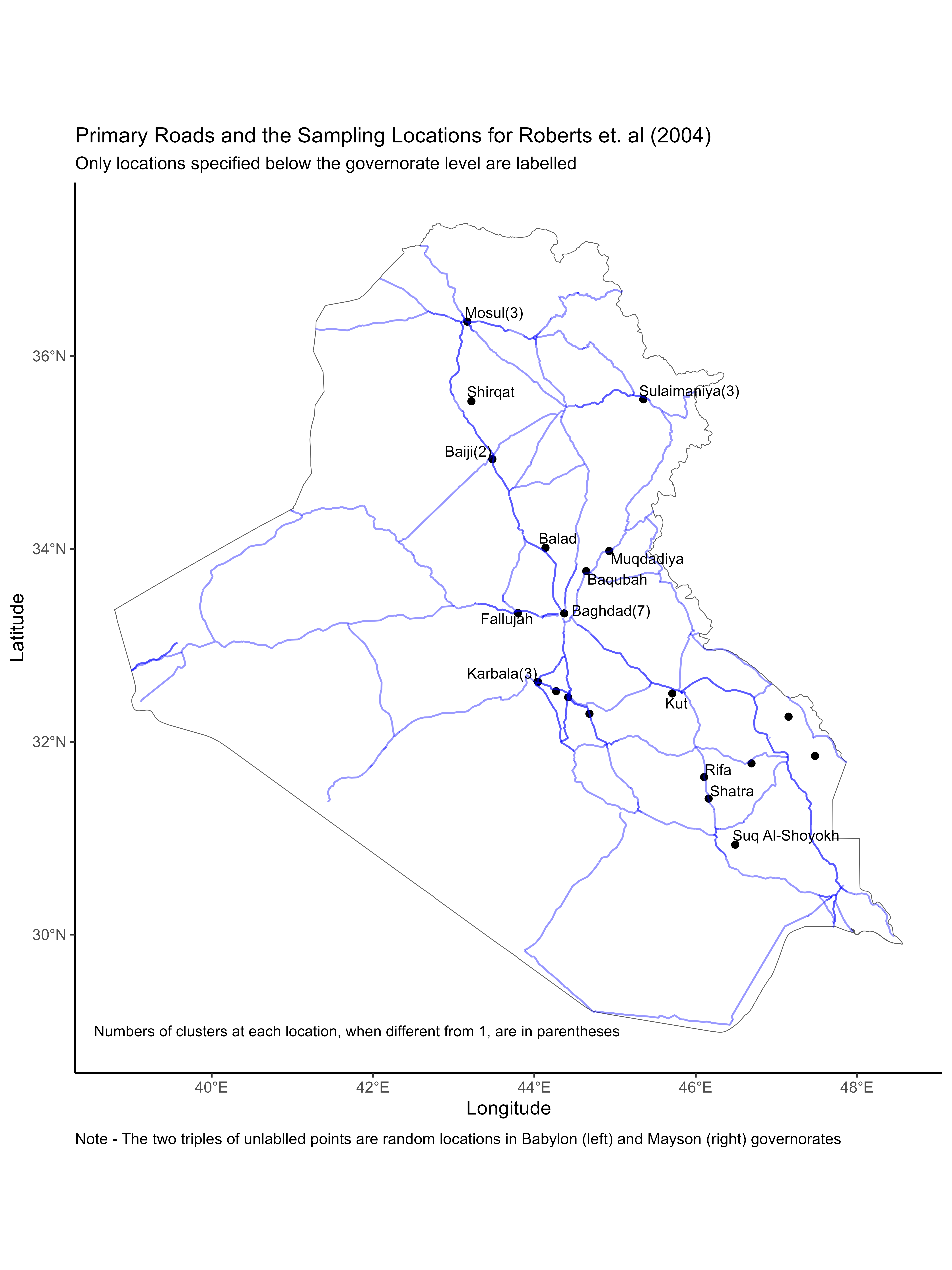

Supplement: S1 Data — (ZIP) [file pone.0297895.s001.zip › figure1.png]

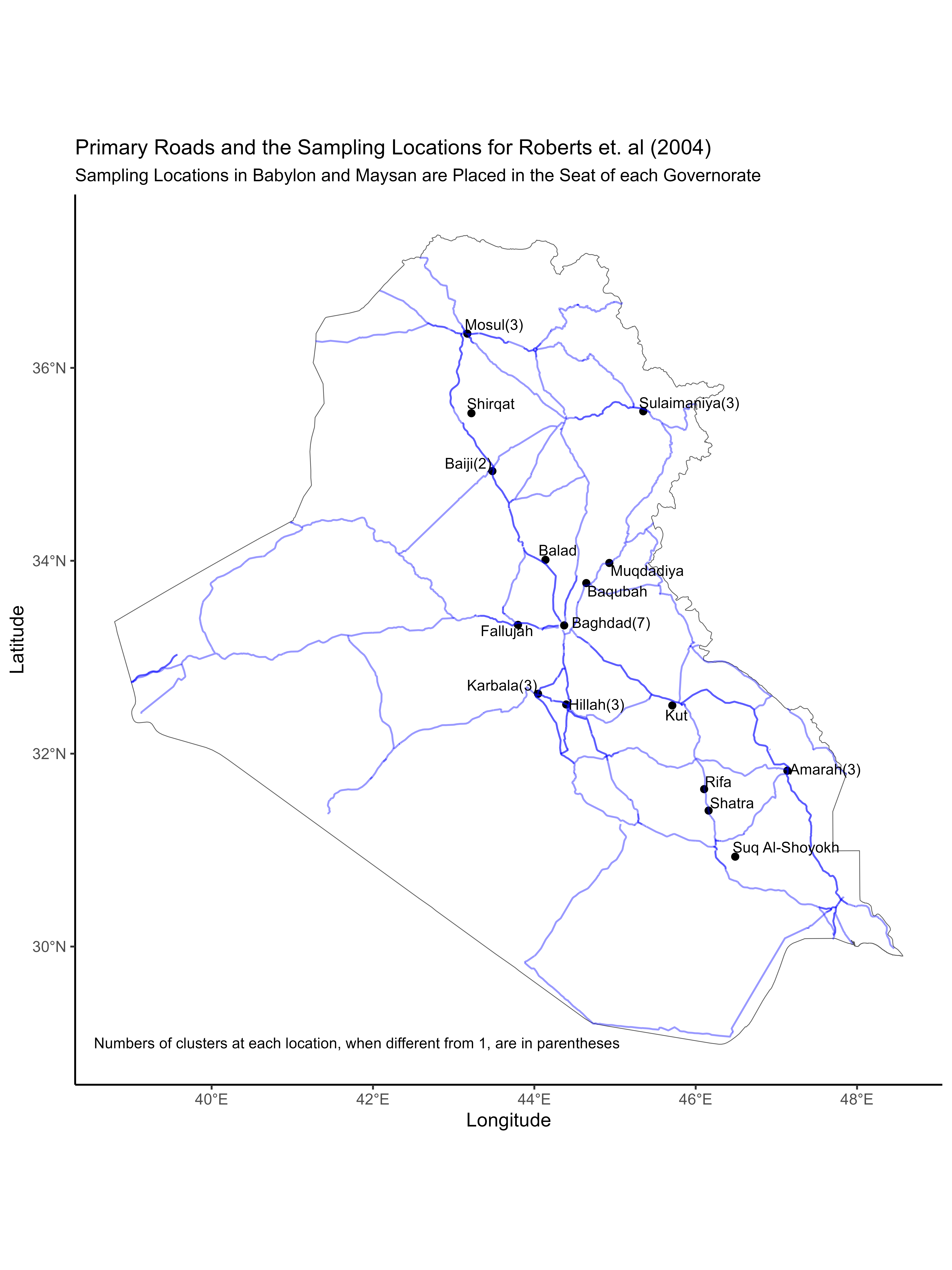

Supplement: S1 Data — (ZIP) [file pone.0297895.s001.zip › figure2.png]

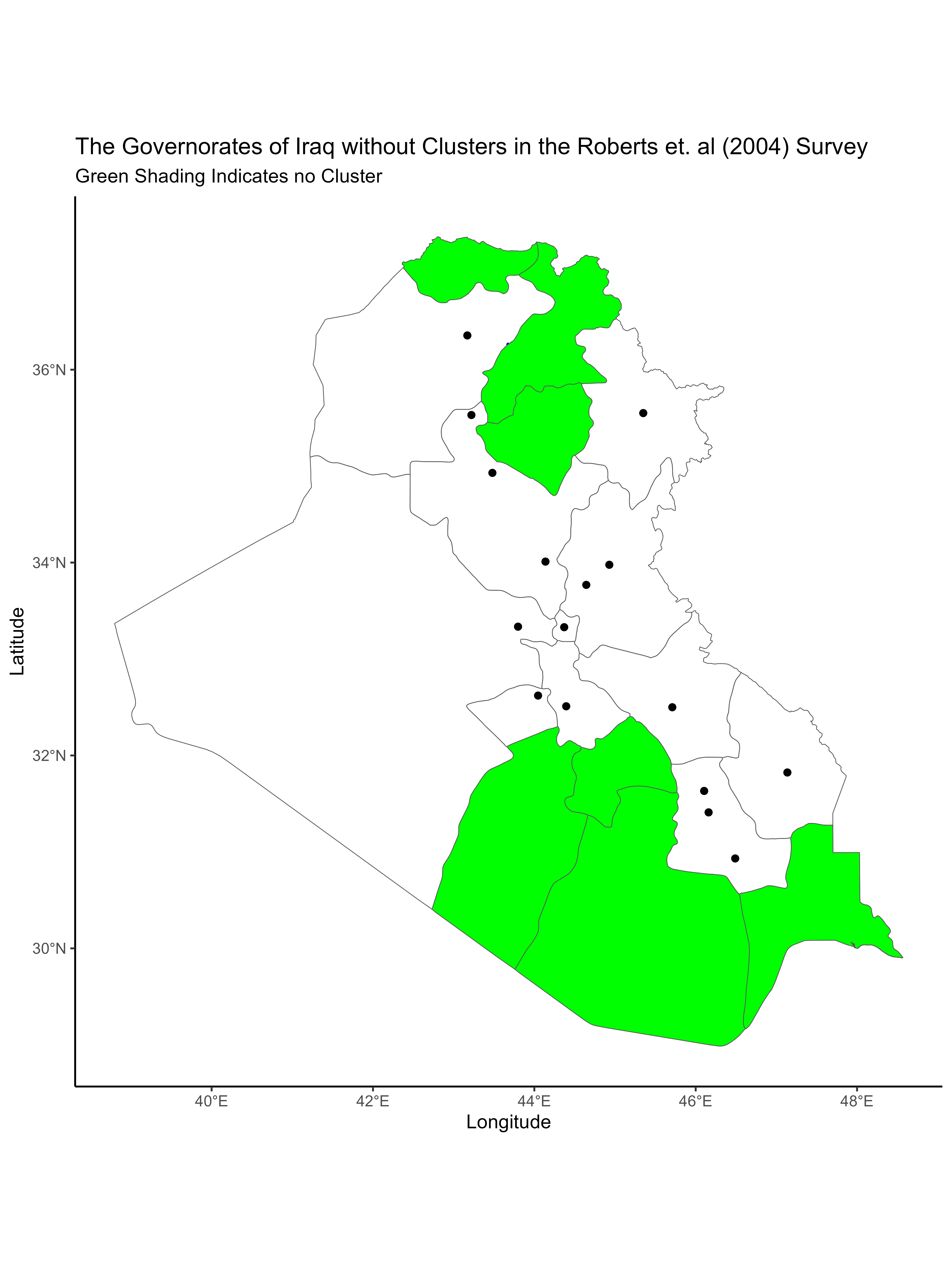

Supplement: S1 Data — (ZIP) [file pone.0297895.s001.zip › figure3.png]

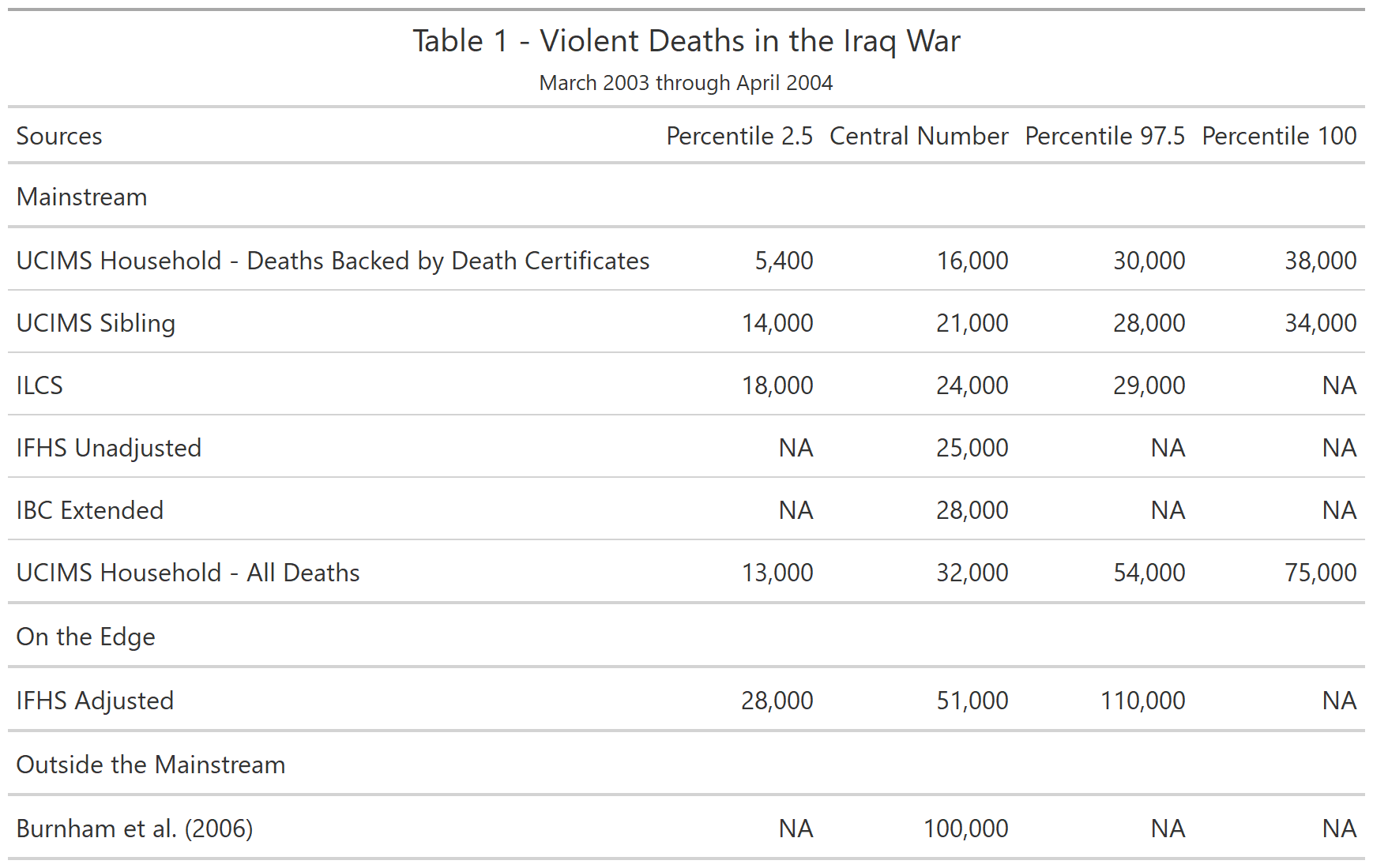

Supplement: S1 Data — (ZIP) [file pone.0297895.s001.zip › table1.png]

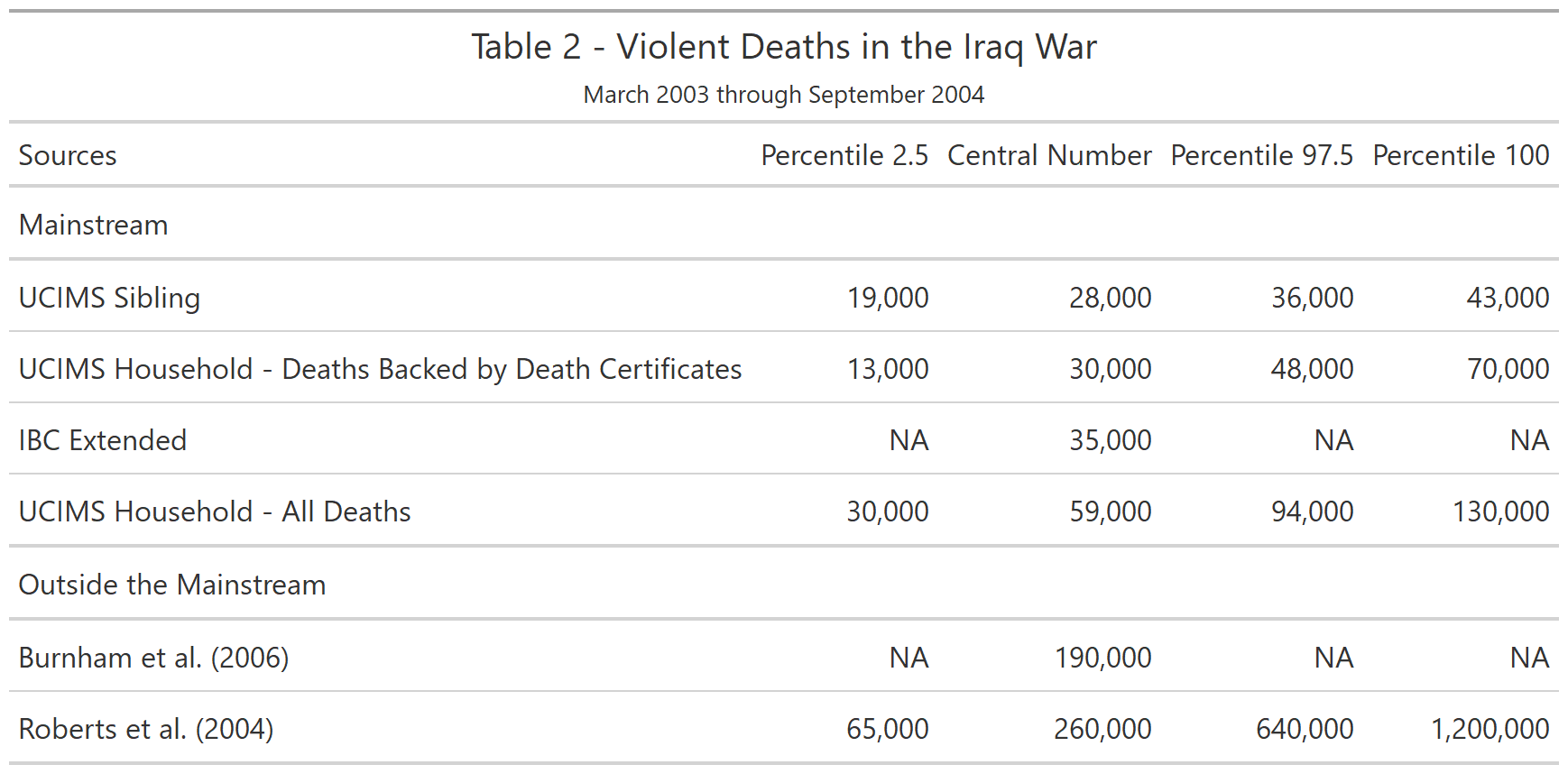

Supplement: S1 Data — (ZIP) [file pone.0297895.s001.zip › table2.png]

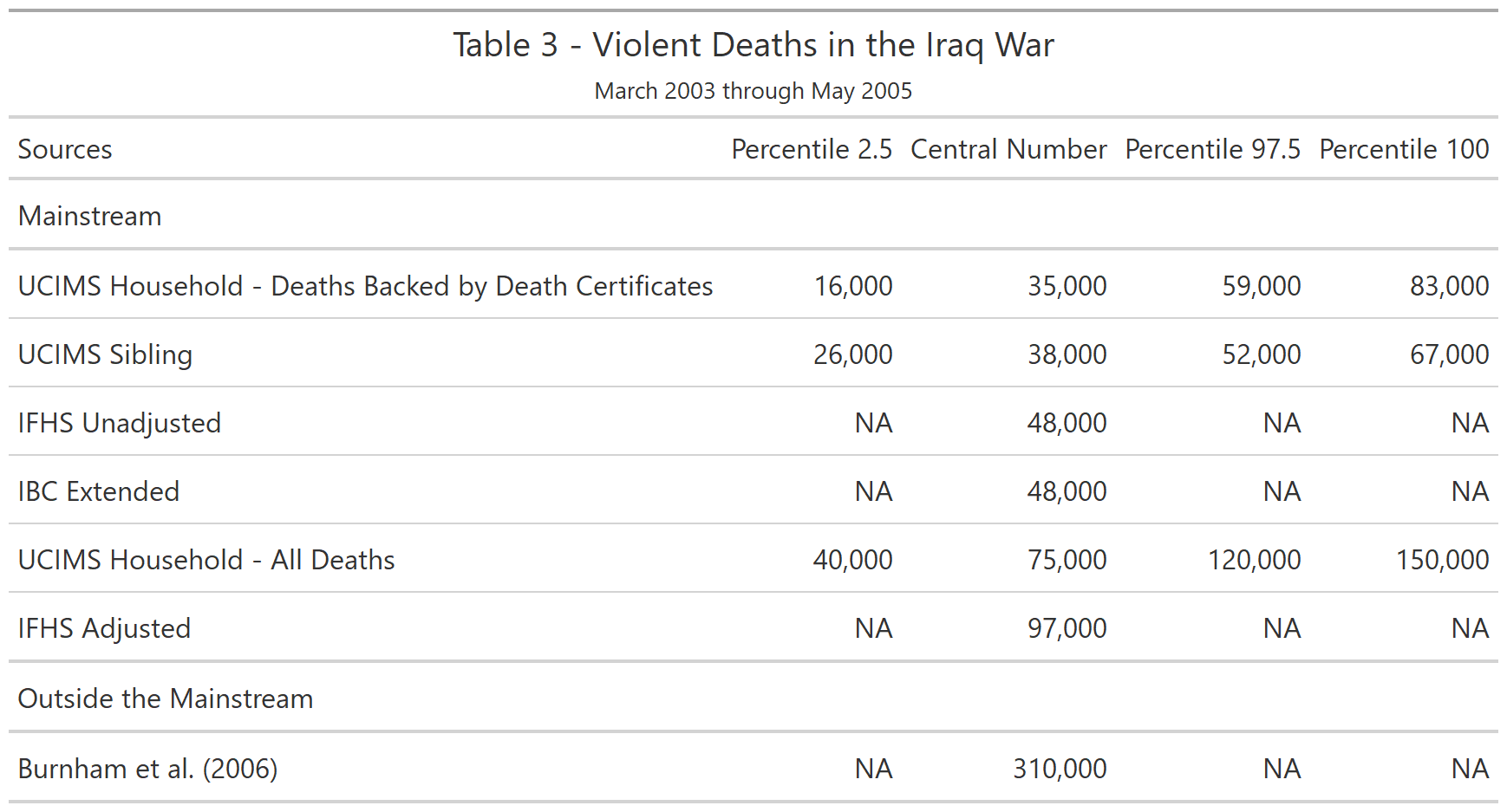

Supplement: S1 Data — (ZIP) [file pone.0297895.s001.zip › table3.png]

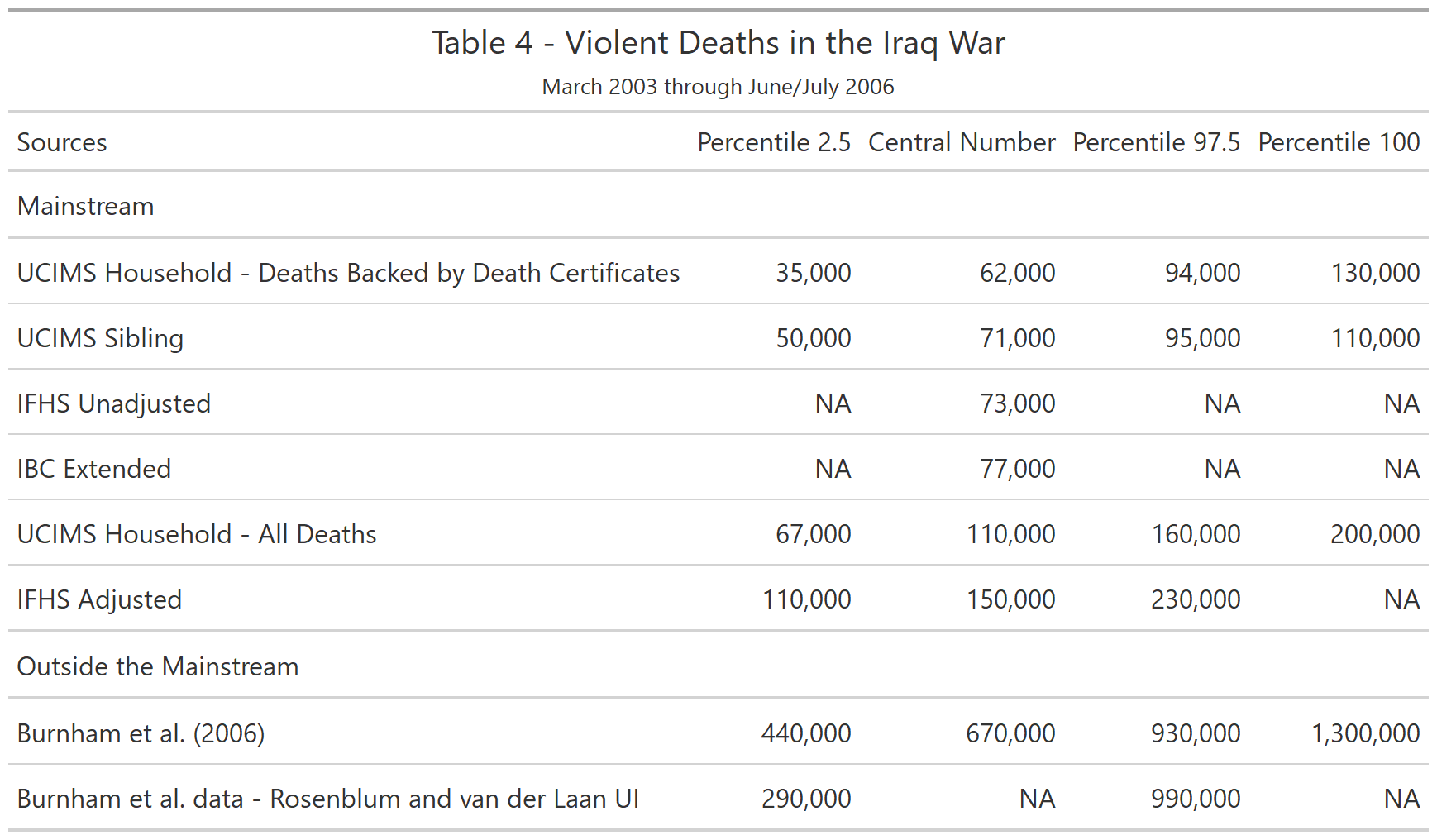

Supplement: S1 Data — (ZIP) [file pone.0297895.s001.zip › table4.png]

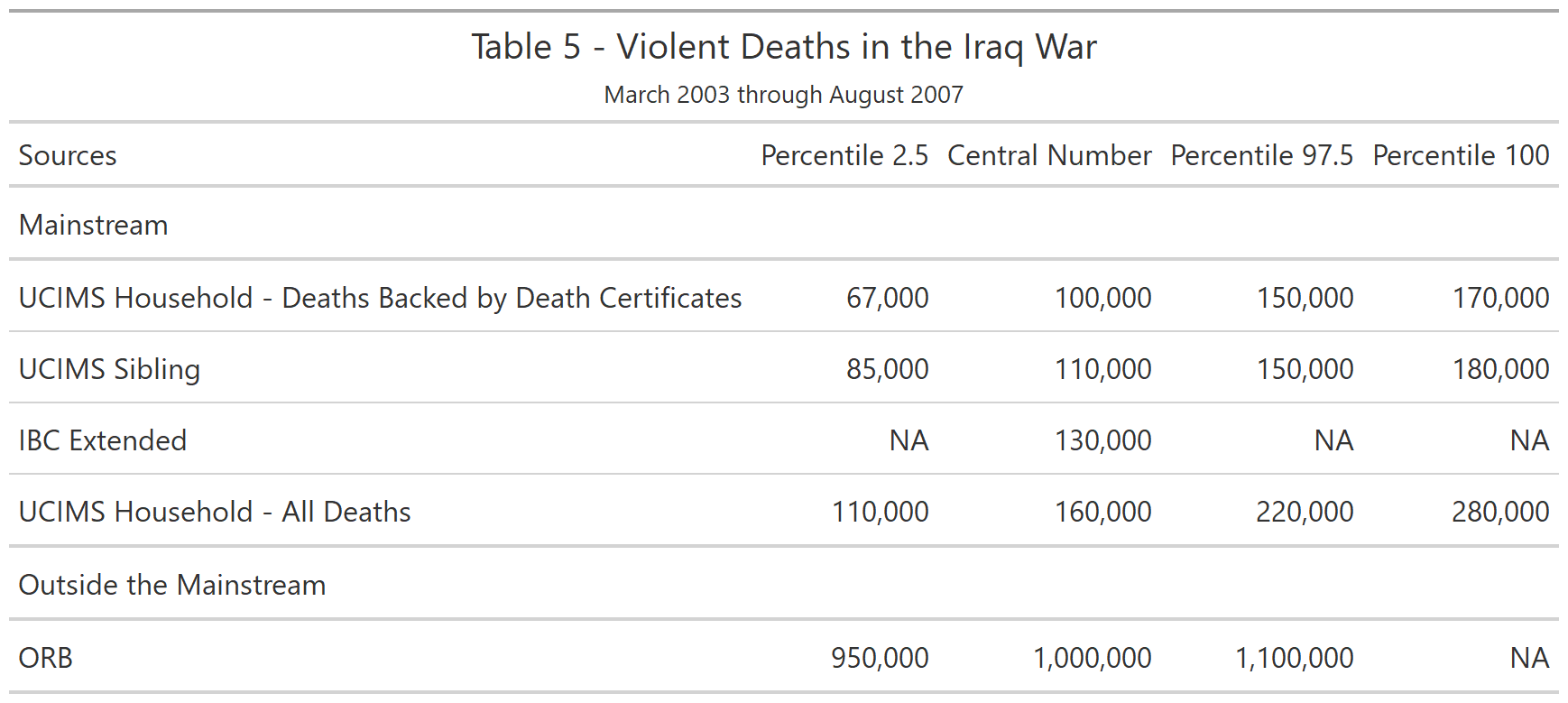

Supplement: S1 Data — (ZIP) [file pone.0297895.s001.zip › table5.png]
