# Supplementary material for: The violent death toll from the Iraq War: 2003–2023
Source: PLoS One. 2024 Feb 27;19(2):e0297895. doi: 10.1371/journal.pone.0297895 (PMC10898744; doi:10.1371/journal.pone.0297895)
Supplement: Attachment — Submitted filename: Comments to Authors.docx [file pone.0297895.s002.docx]

Dear editor I have interestingly read the manuscript and the followings are my suggestions for further improvement to be made by the authors.

In my opinion the study is not suitable for publication in the current form and suggest the followings to be deserved for publishing:

**Abstract**

I would like to add a few comments on the grammar of the abstract:

- In the first sentence, the phrase "right down to the present day" is a bit awkward. It would be better to say "to the present day" or "up to the present day".
- In the second sentence, the phrase "resolves much of the uncertainty" is a bit vague. It would be better to say something like "provides a clearer picture of" or "narrows down the range of uncertainty for".
- In the third sentence, the phrase "all primary sources with coverage that begins in March of 2003" is a bit wordy. It would be better to say "all primary sources that cover the period from March 2003 onwards".

**Introduction**

- The introduction is well-organized and provides a clear overview of the paper's purpose and scope.
- The author does a good job of summarizing the existing literature on the topic and highlighting the gaps in knowledge.
- The author's thesis statement is clear and concise.
- The introduction is grammatically correct and free of errors.

**Methods and Materials**

Here are some specific scientific questions that I have about the Methods and Materials section:

- How did the author account for the different sampling methods used in the different surveys?
- How did the author account for the different biases that may be present in the different surveys?
- How did the author validate the bootstrapping method for the type of data used in this study?
- How did the author choose the data sources for the study?
- How did the author analyze the data?
- What are the limitations of the study?
- How can the findings of the study be generalized to other populations?

**Results**

- Why did the author choose to use the 95% bootstrapped uncertainty intervals in the tables?
- What are the strengths and limitations of the bootstrapping method?
- How did the author account for the different sampling methods used in the different surveys?
- How did the author account for the different biases that may be present in the different surveys?
- How did the author validate the bootstrapping method for the type of data used in this study?
- How confident can we be in the estimates of violent deaths produced by the different surveys?
- What are the implications of the findings of this study for our understanding of the human costs of the Iraq War?

**Discussion**

1. **Comparative Validation:**

How does the rarity of the validation of three distinct methodologies (household-based survey estimation, sibling-based survey estimate, and casualty recording) impact the overall confidence in the study's findings?

Could you elaborate on the significance of the comparable validation found in [35] regarding violent deaths in the Kosovo war? How does this validation contribute to understanding the validity of the methodologies used in this study?

2. **Quantifying Uncertainty:**

How were the 95% uncertainty intervals calculated for individual surveys, and how do these intervals contribute to understanding the remaining uncertainty despite the consistency across group-1 sources?

Could you discuss the reasoning and methodology behind the back-of-the-envelope calculation used to estimate uncertainty for IBC-extended figures post-June 2011? How does this calculation help to address the absence of post-June-2011 surveys?

3. **Validation of IBC Data:**

What factors contribute to the consistent alignment of IBC-extended figures with mainstream sources? How does this consistency bolster the acceptance of IBC-extended numbers as a reliable guide to post-June-2011 violent death tolls?

Considering the change in IBC methodology in July 2011, how was the impact of this change evaluated, and how did it influence the reliability of the IBC-extended numbers?

4. **Quantification and Credibility:**

How does the tentative conclusion regarding the IBC-extended figures' accuracy, drawn from the ratios of IBC-extended figures to group-1 survey central estimates, enhance the credibility of the study's findings?

Could you elaborate on how the median and mean ratios of the back-of-the-envelope calculations contribute to the confidence in the accuracy of the IBC-extended figures?

5. **Project Implications:**

How does the validation of IBC data support the choice of the Costs of War Project to utilize IBC as a basis for quantification of the human cost of the Iraq War and dismiss the group-2 surveys?

In light of the disagreement between [36] and the current study's findings, how does the paper's validation contribute to maintaining credibility in quantification projects, and how can such projects avoid exaggeration?

6. **Holistic Understanding:**

How does the validation of IBC data contribute to the broader understanding of the Iraq War's human cost, as emphasized by [37] and [38], by providing a more accurate representation of individual victims beneath the abstract numbers?

Could you discuss how the validation of IBC data enhances the value of [18]'s casualty recording treatment, and how this approach provides a comprehensive perspective on the Iraq War's impact?
